# Supplementary material for: Dermis resident macrophages orchestrate localized ILC2 eosinophil circuitries to promote non-healing cutaneous leishmaniasis
Source: Nat Commun. 2023 Nov 29;14:7852. doi: 10.1038/s41467-023-43588-2 (PMC10687111; doi:10.1038/s41467-023-43588-2)
Supplement: Supplementary file 1 — Supplementary Information [file 41467_2023_43588_MOESM1_ESM.pdf]

Title: Dermis resident macrophages orchestrate localized ILC2 eosinophil circuitries to promote non healing cutaneous leishmaniasis

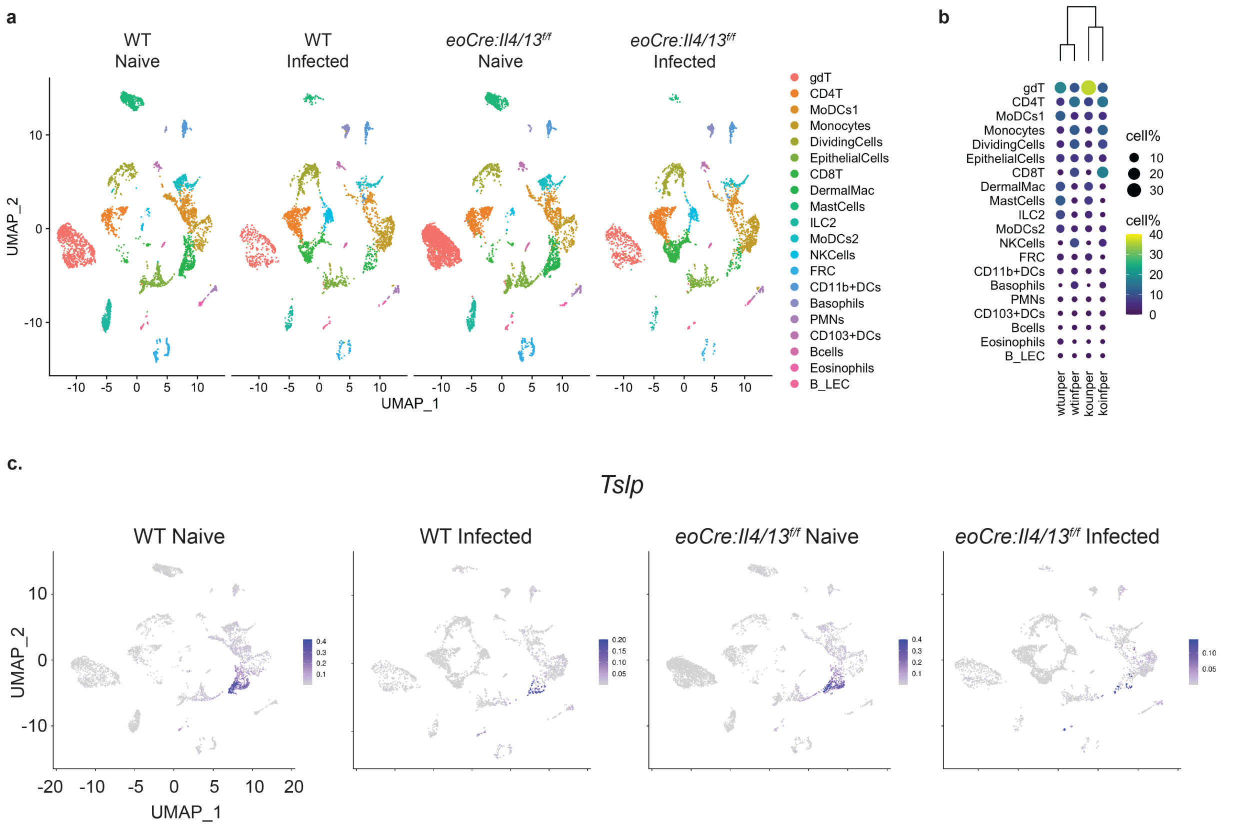

Supplementary Fig. 1. **Single cell transcriptomic analysis of skin isolates from WT and *eoCre : IL4/13*$^{f/f}$ animals with or without LmSd challenge. a** UMAP plots representing of 17,355 cells combined from 4 samples, naïve WT (4,592 cells), naïve *eoCre il4/13*$^{f/f}$ (5,913 cells), infected WT (3,728 cells), and infected *eoCre il4/13*$^{f/f}$ (3,552 cells), 12 days post-challenge with 2 x 10^5 LmSd in the ear dermis. **b** Dot plots visualizing the percentage of cells in clusters in UMAP plots in (A). (cell % is represented 2 ways). **c** UMAP plots of *Tslp* expression in 4 samples.

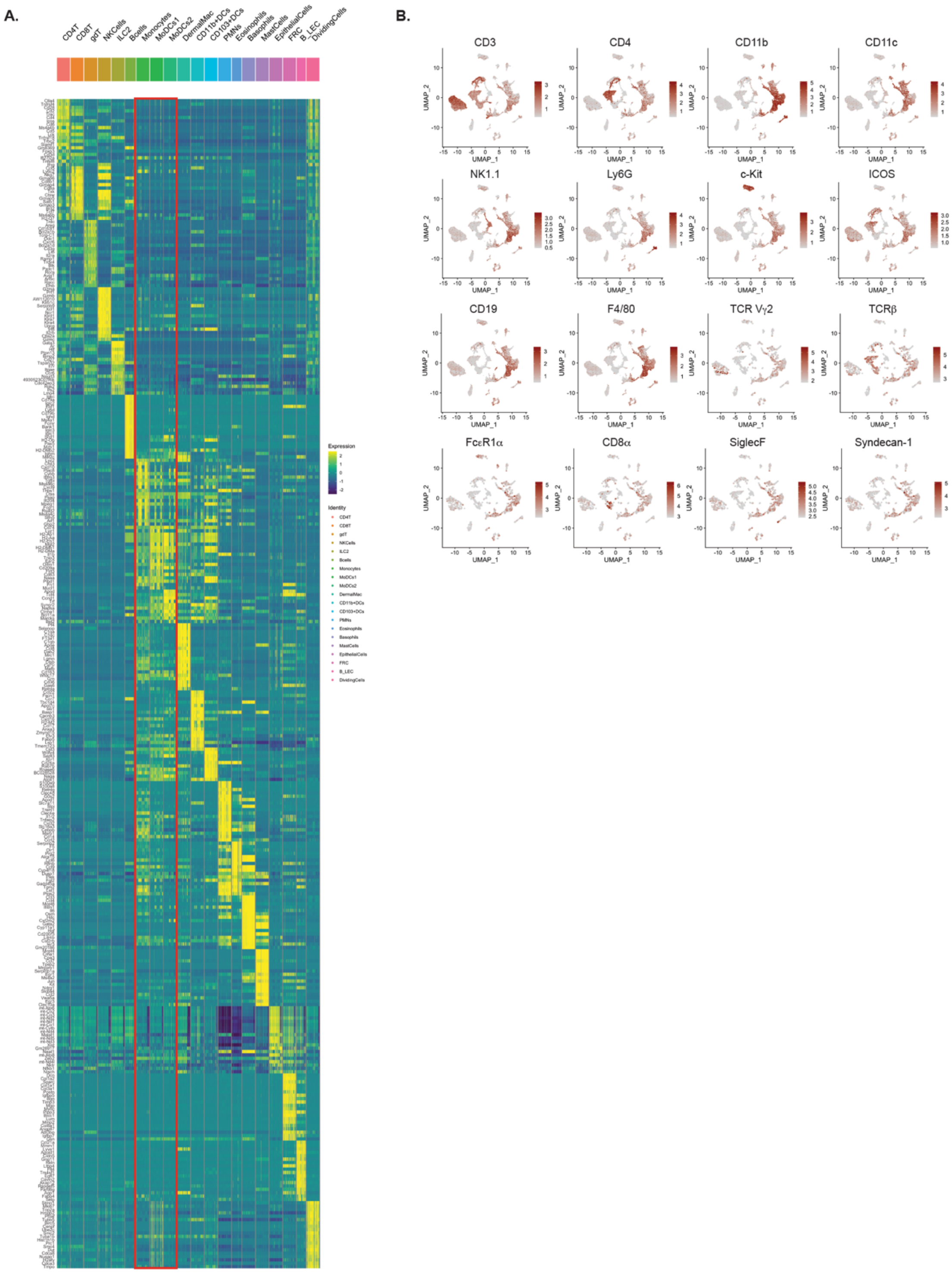

Supplementary Fig. 2. **Transcriptional profiling of 20 scRNA-seq clusters, with accompanying surface marker expression. a**)The heatmap of top 20 DEGs (absolute logFC > 0.5 and p_val_adj < 0.05) of each UMAP cluster shown in Figure 3A. **b** UMAP plots using CITE-Seq to show expression of 16 surface markers.

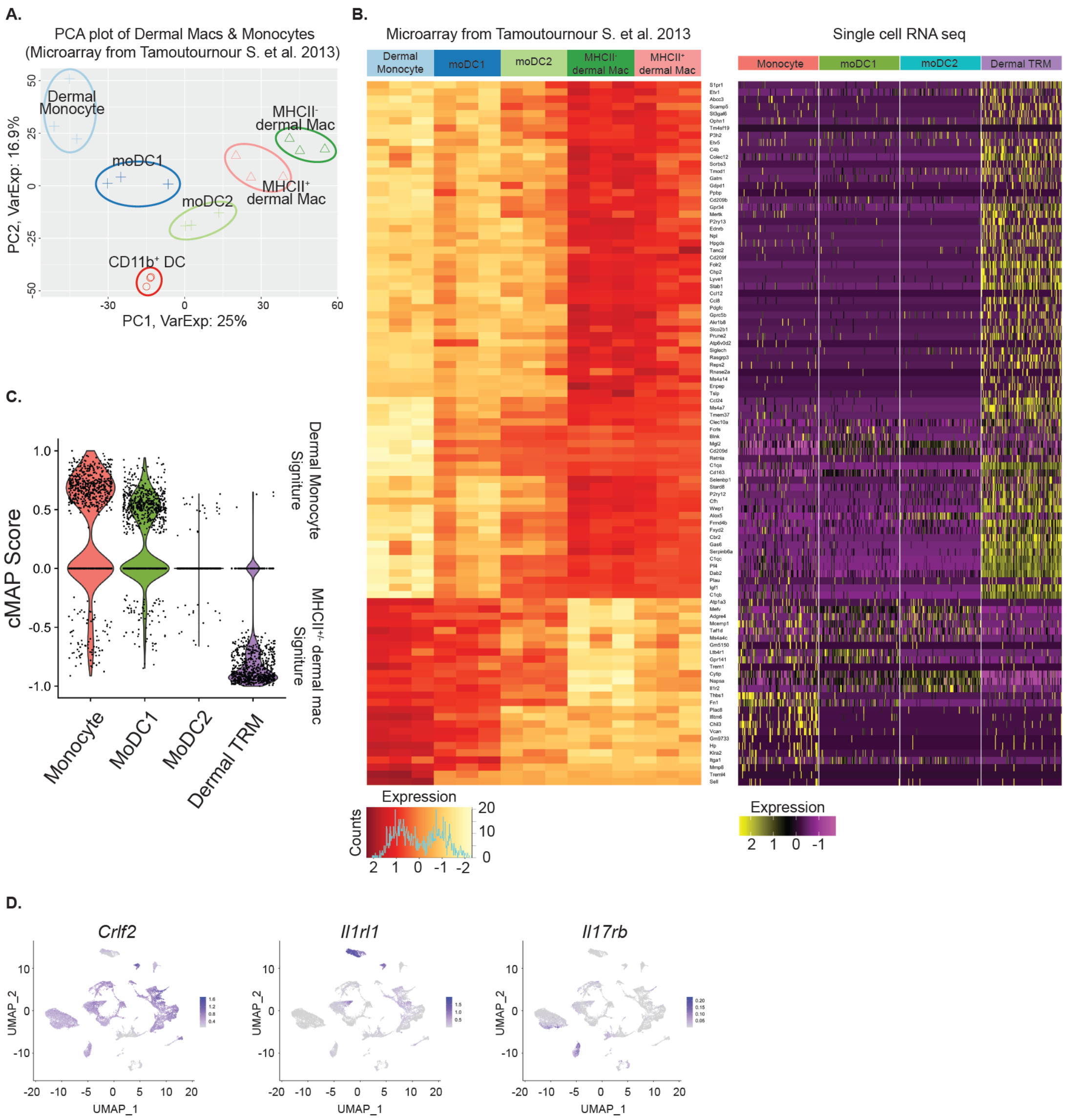

Supplementary Fig. 3. **Identification of 4 myeloid clusters in scRNA-seq. a** Principal component analysis (PCA) of gene expression by the dermal myeloid cells in microarray previously reported [1]. **b** Heatmaps of dermal myeloid cells/clusters from the microarray (left panel) and our scRNA-seq (right panel) based on the expression of 100 DEGs from the comparison between dermal monocyte and MHCII$^{+/-}$ dermal macrophages in the microarray. **c** cMAP analysis of 4 myeloid scRNA-seq clusters showing their enrichment for either dermal monocytes or MHCII$^{+/-}$ dermal macrophage transcriptomes from the microarray. 100 DEGs shown in **b** were used as a reference gene set for this analysis. Cells having transcriptional similarity to neither subset were marked as zero cMAP score. **d** Selected gene expression UMAP plots.

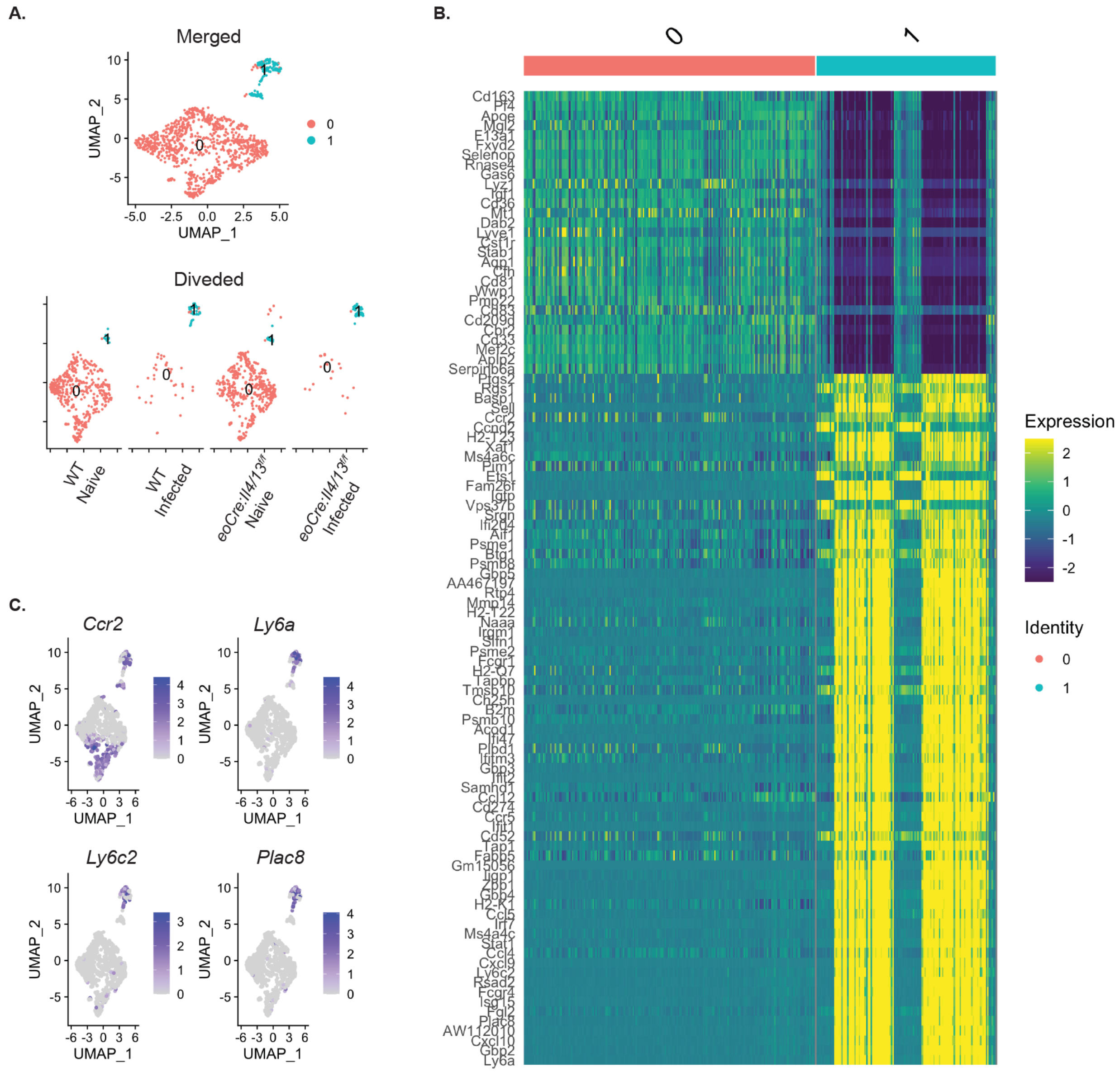

Supplementary Fig. 4. **Separation of monocytic cells from dermal TRM cluster. a** UMAP plots representing the two sub-clusters (cluster 0 and 1) of the original dermal TRM cluster. **b** Heatmap of DEGs between cluster 0 and 1. **c** Selected gene expression UMAP plots.

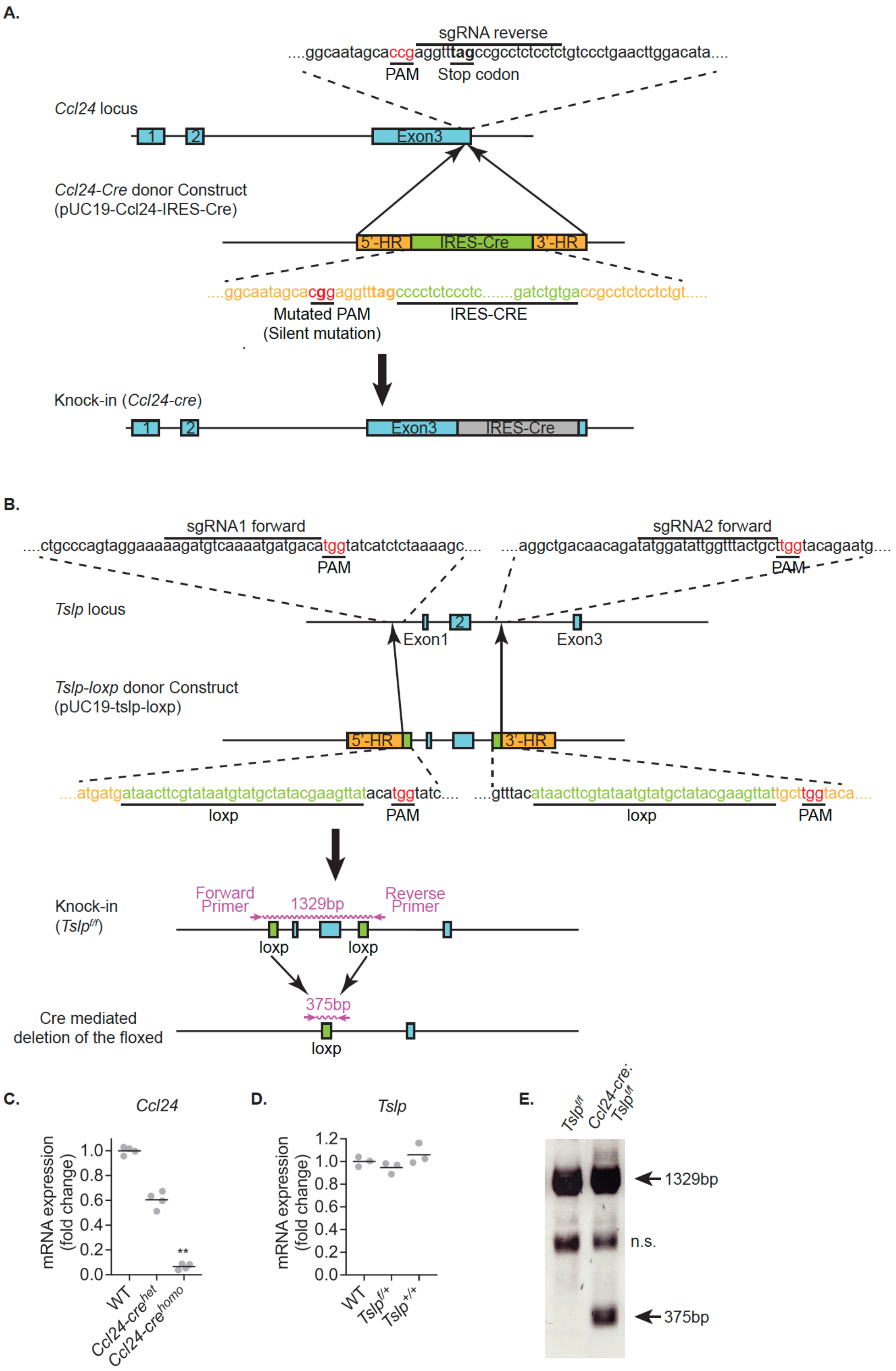

Supplementary Fig. 5. **Generation of *Ccl24-Cre* and *Tslp$^{f/f}$* animals using CRISPR/Cas9 editing. a** Schematic of mouse *Ccl24* locus, targeting construct of *Ccl24-Cre*, and predicted knock-in allele. The translation termination site of the endogenous *Ccl24* gene was linked by an internal ribosomal entry site (IRES) to a Cre element. Blue boxes

denote exons. Sequences are indicated for IRES-Cre (green) and homology arms (orange). The protospacer adjacent motif (PAM) sites are highlighted in red. **b** Schematic of mouse *Tslp* locus, targeting construct of $Tslp^{f/f}$, and predicted knock-in allele. Two loxP sites were designed to surround the first and second exons, whose deletion resulted in a frame-shift mutation in exon3. The green boxes mark loxp sites. PCR screening strategy for cre-mediated deletion of the floxed region was illustrated in magenta. **c** and **d** The quantification of *Ccl24* and *Tslp* mRNA expressions in indicated animals. **e** PCR screening for Cre-mediate deletion of the floxed region in *Ccl24-cre* : $Tslp^{f/f}$ animals based on the strategy in **b**.

A.

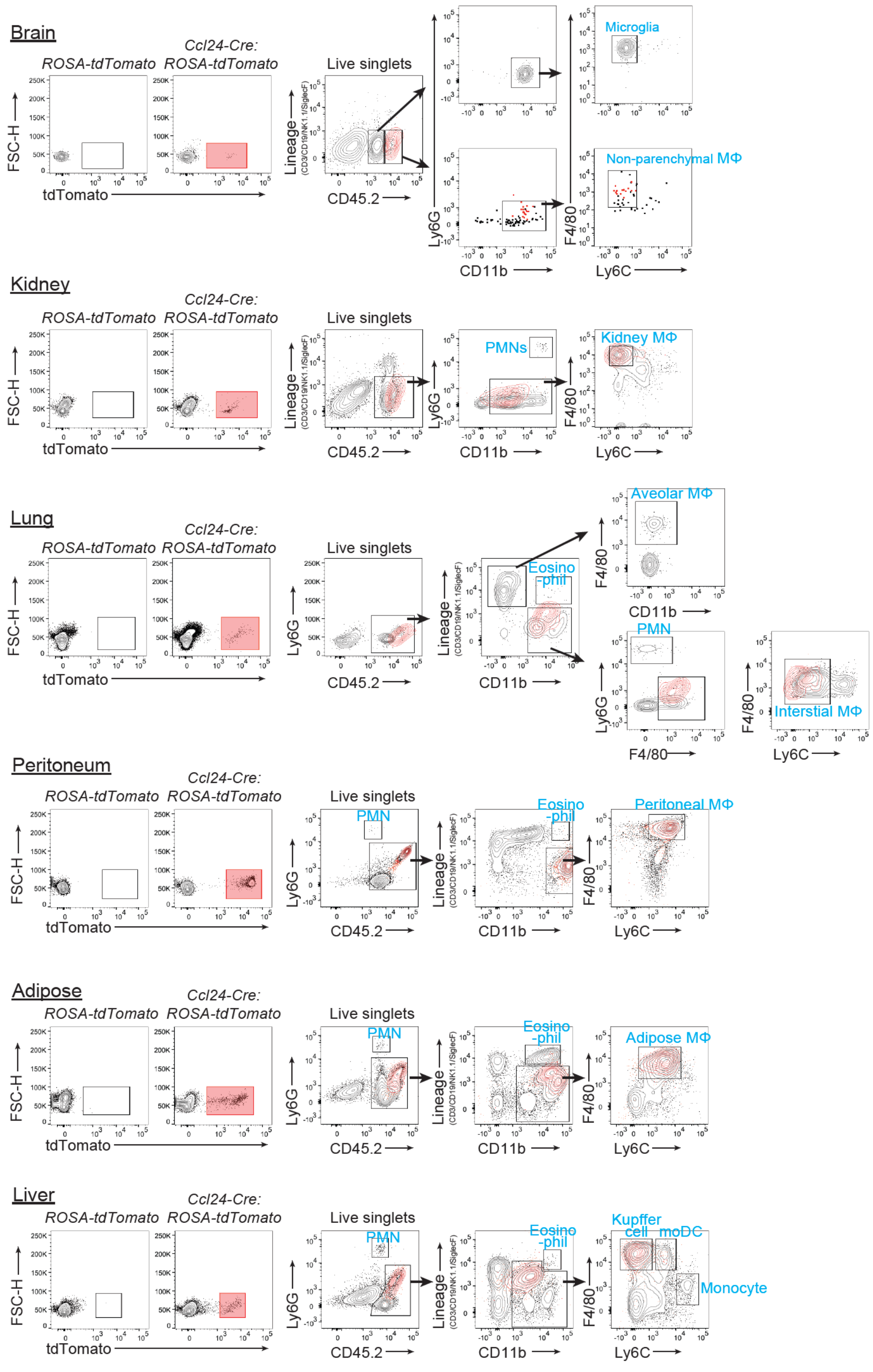

Supplementary Fig. 6. **tdTomato^{+} subsets of TRMs in indicated tissues.** tdTomato^{+} cells in *Ccl24-cre* : *ROSA26-LSL-tdTomato* mice in red were overlayed onto flow-cytometric gatings which identified distinct TRMs from indicated organs.

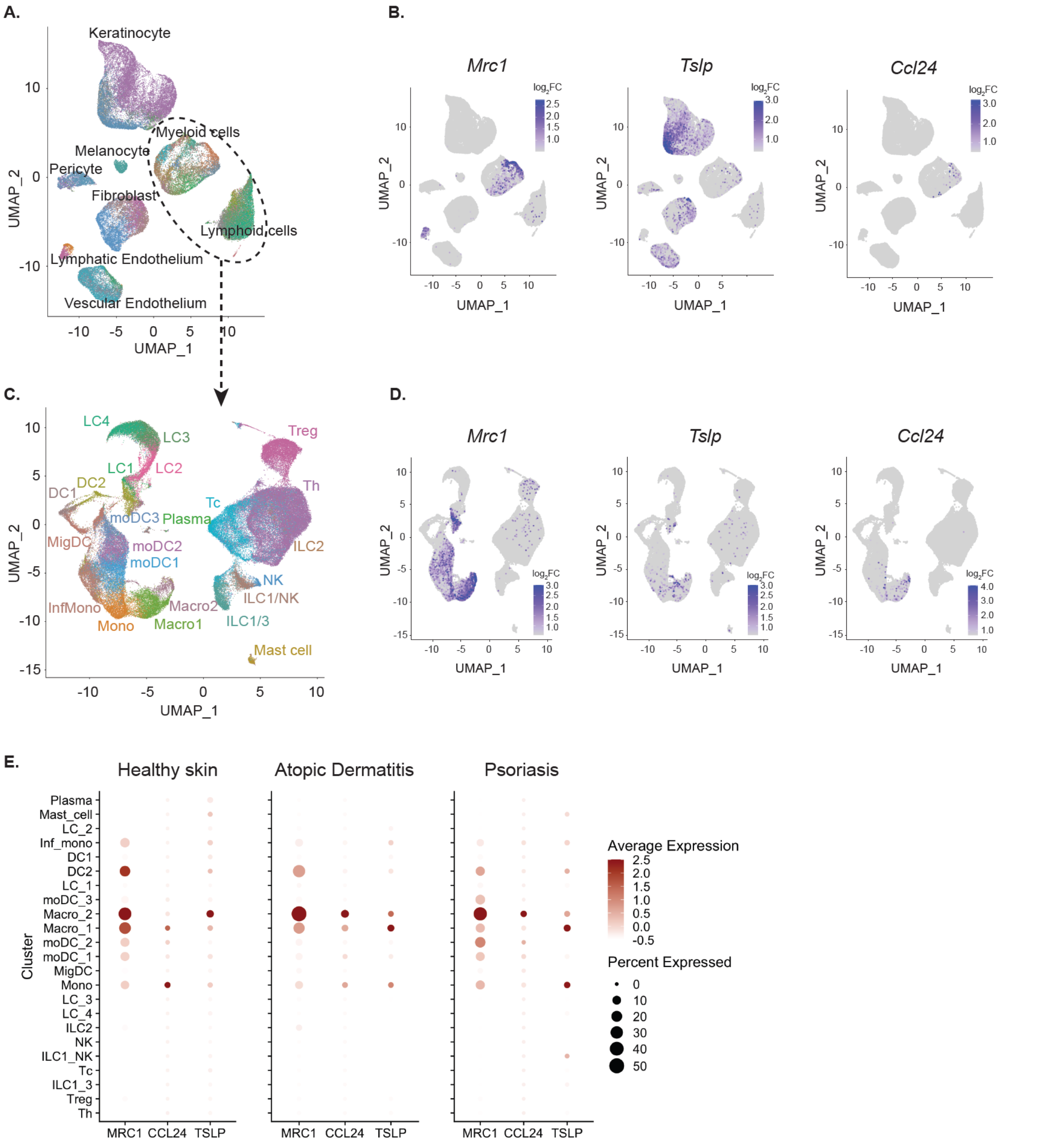

Supplementary Fig. 7. **TRMs co-express *Mrc1*, *Tslp*, and *Ccl24* in healthy and diseased human skin. a** UMAP visualization of human skin samples from the published dataset [2] in 8 major transcriptional clusters of cell types as defined by scRNA-seq analysis. Original single-cell data were down sampled to a total of 59,288 cells from 40 healthy skin, 32 atopic dermatitis (AD), and 24 psoriasis vulgaris (PV) adult human samples. **b** Single-cell expression levels of *Mrc1*, *Tslp* and *Ccl24* represented in UMAP plots in (A). **c** UMAP visualization of 23 different

lymphoid and myeloid cell clusters. **d** Single-cell expression levels of *Mrc1*, *Tslp* and *Ccl24* represented in UMAP plots in (C). **e** Dot plot showing the average expression of *Mrc1*, *Tslp* and *Ccl24* in each cell type relative to all the other cells in the dataset (color bar) and % cells expressing each gene within a cluster (circle size). Cluster annotations presented here are defined as reported[2].

1. Tamoutounour, S. *et al.* Origins and functional specialization of macrophages and of conventional and monocyte-derived dendritic cells in mouse skin. *Immunity* **39**, 925-938 (2013).

2. Reynolds, G. *et al.* Developmental cell programs are co-opted in inflammatory skin disease. *Science* **371** (2021).
