# Supplementary material for: Dermis resident macrophages orchestrate localized ILC2 eosinophil circuitries to promote non-healing cutaneous leishmaniasis
Source: Nat Commun. 2023 Nov 29;14:7852. doi: 10.1038/s41467-023-43588-2 (PMC10687111; doi:10.1038/s41467-023-43588-2)
Supplement: Supplementary file 3 — Reporting Summary [file 41467_2023_43588_MOESM3_ESM.pdf]

nature portfolio

Corresponding author(s): David Sacks

Last updated by author(s): Oct 30, 2023

Reporting Summary

Nature Portfolio wishes to improve the reproducibility of the work that we publish. This form provides structure for consistency and transparency in reporting. For further information on Nature Portfolio policies, see our Editorial Policies and the Editorial Policy Checklist.

Statistics

For all statistical analyses, confirm that the following items are present in the figure legend, table legend, main text, or Methods section.

n/a	Confirmed	
☐	☒	The exact sample size (n) for each experimental group/condition, given as a discrete number and unit of measurement
☐	☒	A statement on whether measurements were taken from distinct samples or whether the same sample was measured repeatedly
☐	☒	The statistical test(s) used AND whether they are one- or two-sided<br>*Only common tests should be described solely by name; describe more complex techniques in the Methods section.*
☒	☐	A description of all covariates tested
☒	☐	A description of any assumptions or corrections, such as tests of normality and adjustment for multiple comparisons
☐	☒	A full description of the statistical parameters including central tendency (e.g. means) or other basic estimates (e.g. regression coefficient) AND variation (e.g. standard deviation) or associated estimates of uncertainty (e.g. confidence intervals)
☐	☒	For null hypothesis testing, the test statistic (e.g. F, t, r) with confidence intervals, effect sizes, degrees of freedom and P value noted<br>*Give P values as exact values whenever suitable.*
☒	☐	For Bayesian analysis, information on the choice of priors and Markov chain Monte Carlo settings
☒	☐	For hierarchical and complex designs, identification of the appropriate level for tests and full reporting of outcomes
☒	☐	Estimates of effect sizes (e.g. Cohen's d, Pearson's r), indicating how they were calculated

Our web collection on statistics for biologists contains articles on many of the points above.

Software and code

Policy information about availability of computer code

Data collection	FACSDiva software {SCR_001456}
Data analysis	FlowJo software v.10.8 {SCR_008520}; Imaris 9.9.1 {SCR_007377}; GraphPad Prism 9.3

For manuscripts utilizing custom algorithms or software that are central to the research but not yet described in published literature, software must be made available to editors and reviewers. We strongly encourage code deposition in a community repository (e.g. GitHub). See the Nature Portfolio guidelines for submitting code & software for further information.

Data

Policy information about availability of data

All manuscripts must include a data availability statement. This statement should provide the following information, where applicable:

- Accession codes, unique identifiers, or web links for publicly available datasets
- A description of any restrictions on data availability
- For clinical datasets or third party data, please ensure that the statement adheres to our policy

The source data for each graph are provided as a Source data file. Single cell RNA sequencing data that support the findings of this study have been deposited in Gene Expression Omnibus (GEO) with the GSE243853 accession number (https://www.ncbi.nlm.nih.gov/geo/query/acc.cgi?acc=GSE243853).

Research involving human participants, their data, or biological material

Policy information about studies with human participants or human data. See also policy information about sex, gender (identity/presentation), and sexual orientation and race, ethnicity and racism.

Reporting on sex and gender	N/A
Reporting on race, ethnicity, or other socially relevant groupings	N/A
Population characteristics	N/A
Recruitment	N/A
Ethics oversight	N/A

Note that full information on the approval of the study protocol must also be provided in the manuscript.

Field-specific reporting

Please select the one below that is the best fit for your research. If you are not sure, read the appropriate sections before making your selection.

- [x] Life sciences
- [ ] Behavioural & social sciences
- [ ] Ecological, evolutionary & environmental sciences

For a reference copy of the document with all sections, see nature.com/documents/nr-reporting-summary-flat.pdf

Life sciences study design

All studies must disclose on these points even when the disclosure is negative.

Sample size	A power analysis was performed to determine the number of animals in groups at 80% power and type 1 error of 5% based on standard deviations from our previous studies.
Data exclusions	None of data were excluded.
Replication	All of data were successfully replicated at least two times. The number of replication is indicated in each figure legend.
Randomization	The experimental groups were distributed across multiple cages and litters, and the location of the mouse cages were randomized on the racks.
Blinding	All the investigators were blinded to group allocation for measuring lesion development and acquiring samples from animals.

Reporting for specific materials, systems and methods

We require information from authors about some types of materials, experimental systems and methods used in many studies. Here, indicate whether each material, system or method listed is relevant to your study. If you are not sure if a list item applies to your research, read the appropriate section before selecting a response.

Materials & experimental systems

n/a	Involved in the study	
[ ]	[x]	Antibodies
[x]	[ ]	Eukaryotic cell lines
[x]	[ ]	Palaeontology and archaeology
[ ]	[x]	Animals and other organisms
[x]	[ ]	Clinical data
[x]	[ ]	Dual use research of concern
[x]	[ ]	Plants

Methods

n/a	Involved in the study	
[x]	[ ]	ChIP-seq
[ ]	[x]	Flow cytometry
[x]	[ ]	MRI-based neuroimaging

Antibodies

Antibodies used	PE anti-mouse CD90.2 (53-2.1, Cat#140307, Biolegend); PE/Cy7 anti-mouse CD2 (RM2-5, Cat#100113, Biolegend); APC anti-mouse CD3 (17A2, Cat#100235, Biolegend); Alexa488 anti-mouse TCRb (H57-597, Cat#109216, Biolegend); PerCP/Cy5.5 anti-mouse TCRg/d (GL3, Cat#118117, Biolegend); FITC, APC/Cy7, and Brilliant VioletTM 421 anti-mouse Ly6G (1A8, Cat#127605/127623/127627, Biolegend); APC/Cy7 anti-mouse Ly6C (HK1.4, Cat#128025, Biolegend); FITC and PE/Cy7 anti-mouse CD11b (M1/70, Cat#101205/101215, Biolegend); PerCP/Cy5.5, Brilliant VioletTM 421, and APC/Cy7 anti-mouse NK1.1 (PK136,

	Cat#108727/108731/108723, Biolegend); Alexa647 and APC anti-mouse CD206 (C068C2, Cat#141711/141707, Biolegend); PE and Brilliant VioletTM 421 anti-mouse Siglec-F (s17007L, Cat#155505/155509, BD Biosciences); PerCP/Cy5.5 anti-mouse CD45.2 (104, Cat#109827, Biolegend); APC and APC/Cy7 anti-mouse F4/80 (BM8, Cat#123115/123117, Biolegend); Brilliant VioletTM 421 anti-mouse IL-5 (TRFK5, Cat#504311, Biolegend); Alexa488 anti-mouse IL-13 (eBio13A, Cat#12-7133-41, Thermo-Fisher Scientific); eFluor450 anti-mouse CD31(390, Cat#48-0311-82, Invitrogen); PE anti-mouse CD45.2 (104, Cat#109808, Biolegend); TotalSeqTM-B0182 anti-mouse CD3 (17A2, Cat#100257, Biolegend); TotalSeqTM-B0001 anti-mouse CD4 (RM4-5, Cat#100573, Biolegend); TotalSeqTM-B0014 anti-mouse CD11b (M1/70, Cat#101273, Biolegend); TotalSeqTM-B0106 anti-mouse CD11c (N418, Cat#117359, Biolegend); TotalSeqTM-B0093 anti-mouse CD19 (6D5, Cat#115563, Biolegend); TotalSeqTM-B0118 anti-mouse NK1.1 (PK136, Cat#108763, Biolegend); TotalSeqTM-B0015 anti-mouse Ly6G (1A8, Cat#127659, Biolegend); TotalSeqTM-B0012 anti-mouse c-Kit (2B8, Cat#105849, Biolegend); TotalSeqTM-B0847 anti-mouse ICOS (7E.17G9, Cat#117425, Biolegend); TotalSeqTM-B0114 anti-mouse F4/80 (BM8, Cat#123155, Biolegend); TotalSeqTM-B0157 anti-mouse CD45.2 (104, Cat#109859, Biolegend); TotalSeqTM-B0115 anti-mouse FceR1a (MAR-1, Cat#134341, Biolegend); TotalSeqTM-B0002 anti-mouse CD8a (53-6.7, Cat#100783, Biolegend); TotalSeqTM-B0431 anti-mouse SiglecF (S17007L, Cat#155517, Biolegend); TotalSeqTM-B0810 anti-mouse CD138 (281-2, Cat#142536, Biolegend); TotalSeqTM-B0120 anti-mouse TCRb (H57-597, Cat#109261, Biolegend); TotalSeqTM-B0211 anti-mouse TCRgd2 (UC3-10A6, Cat#137715, Biolegend); TotalSeqTM-B0301 to 0304 anti-mouse Hashtag 1 to 4 (M1/42 and 30-F11, Cat#155831/155833/155835/155837, Biolegend)
Validation	All the antibodies are in common use for immunological analysis and have been validated by the manufacturer as follows; 1) Testing on multiple cell and tissue types with a variety of known expression levels. 2) Validation in multiple applications as a cross-check for specificity and to provide additional clarity for researchers. 3) Comparison to existing antibody clones. 4) Using cell treatments to modulate target expression, such as phosphatase treatment to ensure phospho-antibody specificity. The details of validation methods and materials are also available on respective manufacturer home pages for each antibody.

Animals and other research organisms

Policy information about studies involving animals; ARRIVE guidelines recommended for reporting animal research, and Sex and Gender in Research

Laboratory animals	C57BL/6NTAC, Il25-/-, Il33-/-, B6(C)-Il5tm1.1(icre)Lky/J, ROSA26-LSL-tdTomato, C57BL/6-Gt(ROSA)26Sortm1(HBEGF)Awai/J, Gata3tm1.1Mbu/J, Tslpr-/-, Il4-/-, eoCre, Il4/13f/f, Ccl24-Cre, and Tslpf/f mice were used in this study. All the mice used in these studies were female, 6-8 weeks old, and were bred and maintained in the NIAID animal care facility under specific pathogen-free condition and at a constant cycle of 14 h in the light and 10 h in the dark. Colonies were maintained at 72 +/- 3 °F and 30–70% humidity, with free access to food and water.
Wild animals	This study did not involve wild animals.
Reporting on sex	All experiments used female mice.
Field-collected samples	This study did not involve samples collected in the field.
Ethics oversight	They were used under a study protocol approved by the NIAID Animal Care and Use Committee (protocol number LPD 68E)

Note that full information on the approval of the study protocol must also be provided in the manuscript.

Flow Cytometry

Plots

Confirm that:

- [x] The axis labels state the marker and fluorochrome used (e.g. CD4-FITC).
- [x] The axis scales are clearly visible. Include numbers along axes only for bottom left plot of group (a 'group' is an analysis of identical markers).
- [ ] All plots are contour plots with outliers or pseudocolor plots.
- [x] A numerical value for number of cells or percentage (with statistics) is provided.

Methodology

Sample preparation	Single-cell suspensions were stained with LIVE/DEAD Fixable Aqua Dead Cell Stain Kit (Thermofisher) and incubated with an anti-Fcγ II/III (CD16/32) receptor Ab (2.4G2, BD Biosciences) in PBS containing 1% FCS followed by fluorochrome-conjugated antibodies for 1 hour on ice. For intracellular detection of cytokines, single cell suspension harvested from tissues were cultured in the presence of PMA/Ionomycin and Brefeldin A (Biolegend) for 4 h at 37°C. The staining of surface and cytoplasmic cytokines/chemokine was performed sequentially. Cells were first incubated with LIVE/DEAD Fixable Aqua Dead Cell Stain Kit (Thermofisher). They were stained for their surface markers, then fixed and permeabilized using BD Cytofix/Cytoperm (BD Biosciences), and finally stained for detection of cytokines/chemokine for 30 min on ice.
Instrument	FacsCANTO II flow cytometer (BD Biosciences)
Software	FlowJo software (Tree Star)
Cell population abundance	The sorting purity of CD45+, CD45-, and ILC2 was more than 95% which was checked by post-sort running.

Gating strategy	Flow Cytometry following infection : Cell were first gated on FSC-A vs SSC-A out from debris, and doublets were further excluded through FSC-H vs FSC-W. Dead cells were excluded by Live/Dead Fixable Aqua staining. After myeloid cells were gated on CD11b+, eosinophils and neutrophils were identified as SiglecF+ and Ly6G+ respectively. Dermal TRMs, monocytes and moDCs were gated out of remaining CD11b+SiglecF-Ly6G- population as follows; MRhi, Ly6Chi MRlow, Ly6Cint-low MRlow. Gating strategies for ILC2s and TRMs from other tissues are provided in Figure 1a and sFig 6.

☒ Tick this box to confirm that a figure exemplifying the gating strategy is provided in the Supplementary Information.
